# Phthalates, Para-Hydroxybenzoic Acids, Bisphenol-A, and Gonadal Hormones’ Effects on Susceptibility to Attention-Deficit/Hyperactivity Disorder

**DOI:** 10.3390/toxics8030057

**Published:** 2020-08-13

**Authors:** Ching-Shu Tsai, Wen-Jiun Chou, Sheng-Yu Lee, Min-Jing Lee, Miao-Chun Chou, Liang-Jen Wang

**Affiliations:** 1 Department of Child and Adolescent Psychiatry, Kaohsiung Chang Gung Memorial Hospital and Chang Gung University College of Medicine, Kaohsiung 833401, Taiwan; jingshutsai@yahoo.com.tw (C.-S.T.); wjchou@cgmh.org.tw (W.-J.C.); chuncat188@gmail.com (M.-C.C.); 2 Department of Psychiatry, Kaohsiung Veterans General Hospital, Kaohsiung 813414, Taiwan; shirleylee.ncku@gmail.com; 3 Department of Psychiatry, College of Medicine, Graduate Institute of Medicine, School of Medicine, Kaohsiung Medical University, Kaohsiung 807378, Taiwan; 4 Department of Child and Adolescent Psychiatry, Chia-Yi Chang Gung Memorial Hospital, Chia-Yi City 613016, Taiwan; 8035c@cgmh.org.tw

**Keywords:** ADHD, endocrine disrupting chemicals, gonadal hormones, sex hormone-binding globulin, cognition

## Abstract

This study aimed to examine whether endocrine-disrupting chemicals (EDCs), such as phthalates, para-hydroxybenzoic acids, and bisphenol-A (BPA), affect gonadal hormones and further link to the susceptibility to attention-deficit/hyperactivity disorder (ADHD). We recruited 98 boys with ADHD, 32 girls with ADHD, 42 boys without ADHD and any other psychiatric disorders, and 26 girls without ADHD and any other psychiatric disorders. Urine levels of EDCs, including mono-methyl phthalate (MMP), monoethyl phthalate (MEP), mono-n-butyl phthalate (MnBP), monobenzyl phthalate (MBzP), monoethylhexyl phthalate (MEHP), methylparaben (MP), ethylparaben (EP), propylparaben (PP), butylparaben (BP), and bisphenol A (BPA), were examined. Endocrine systems were evaluated by using the serum levels of follicle-stimulating hormone (FSH), luteinizing hormone (LH), testosterone, free testosterone, estradiol, progesterone, sex hormone-binding globulin (SHBG), and prolactin. We found that boys with ADHD had higher levels of MnBP and EP than control boys. There were no significant differences regarding EDCs between the females with ADHD and control groups. No significant differences in testosterone, free testosterone, FSH, LH, estradiol, progesterone, or SHBG were found between the ADHD group and controls among either boys or girls. Among boys with ADHD, urine MBzP and MEHP levels were positively correlated with serum testosterone levels. Among girls, urine MEP levels were positively correlated with serum LH, testosterone, and free testosterone levels. The findings suggest that the possibility of an adverse impact of EDCs on gonadal hormones and neurodevelopment may exist. However, the results could be subject to potential selection bias, and the findings in this study should be interpreted with caution.

## 1. Introduction

Attention-deficit/hyperactivity disorder (ADHD) is among the most common psychiatric disorders among school-aged children and adolescents. The prevalence of ADHD of school-age children are estimated as 5–10% [1,2,3]. ADHD patients suffered from functional impairments in academic, occupational, and social contexts throughout childhood, adolescence, and adulthood [4,5,6]. Although twin, adoption, and molecular genetic studies have shown that ADHD is highly inherited, a number of environmental factors may be involved in susceptibility to ADHD [7]. For example, organic pollutants (e.g., pesticides, polychlorinated biphenyl (PCBs)) and lead have been considered as increasing the risk of ADHD [8]. Studies have indicated that PCB exposure leads to impairments in cognitive flexibility and response inhibition, which are comparable with those observed in ADHD [9]. Exposure to environmental chemicals or toxics is one of those important environmental factors.

Endocrine-disrupting chemicals (EDCs) are chemicals that can interfere with endocrine (or hormonal) systems at certain doses [10]. EDCs can negatively affect the development of a child, including gonadal hormones, pubertal development, and neurodevelopment [11]. Exposure to EDCs can alter or eliminate sexually dimorphic behaviors, and the disruptions of such behaviors may be detrimental to social adaptation and response capability [12]. ADHD exhibits greater burden of risk in male individuals and has been proposed to be associated with disrupted developmental trajectory and the sexual dimorphism of brain maturation [13,14]. Previous human studies have demonstrated that exposure to EDCs was related to such neurodevelopmental disorders as decreased intelligence, poorer memory, autism spectrum disorders, ADHD, and other behavioral problems [10]. However, the relationships between perinatal exposure to EDCs in humans and ADHD have not been consistent among previous studies [15].

Among the EDCs, phthalates, para-hydroxybenzoic acid (paraben), and bisphenol A (BPA) had been reported to exert neuropsychiatric adverse effects. A case-control study conducted in China revealed that child exposure to phthalates may contribute to ADHD and related behavior problems [16]. A recent study indicates that methylparaben in meconium was associated with preterm birth, decreased gestational age, and risk of developing ADHD [17]. Several studies had demonstrated associations between BPA levels and increased risk of ADHD [18,19]. However, a previous review article indicated no firm conclusion regarding the association between ADHD and numerous environmental factors such as phthalates, BPA, polycyclic aromatic hydrocarbons (PAHs), and polyfluoroalkyl chemicals (PFCs) [20]. In sum, phthalates, parabens, and BPA are a substantial health concern; but the relationships between these EDCs and ADHD warrant further investigation.

In addition to the impact of EDCs, gonadal hormones also play a major role in the organization of the brain and behavioral systems [21]. Gonadal hormones not only influence sexual behavior, but are also involved in cognitive function, mood, and socialization [22]. The gonadal hormone accounts for most of the known sex differences in neural structure and behavior via upstream influences on brain organization and plasticity and downstream effects on behavioral expression [23]. Prenatal testosterone may modulate the organization of dopaminergic circuits and dopamine function, putting boys at a higher risk of developing inattention and disruptive behavioral disorders [14]. In contrast, the activating effects of estradiol at puberty may regulate the amygdala and other circuits, particularly the effects on the serotonergic pathway, thus placing girls at a greater risk of internalization and mood disorders [14]. An animal study has shown that the concentrations of progesterone were significantly elevated in 10-week-old hypertensive rats when compared to 10-week-old normotensive rats [24]. Compared to age- and sex-matched healthy control boys, prepubertal, stimulant-naïve boys with ADHD had significantly lower sex-hormone-binding globulin (SHBG) and higher free and bioavailable testosterone percentages [25]. One cross-sectional study revealed that free testosterone level did not have a significant correlation with ADHD clinical symptoms, but SHBG levels were found to be negatively correlated with ADHD behavior symptoms among boys [26]. Another study revealed that decreased levels of estradiol in the context of increased levels of either progesterone or testosterone were associated with more severe ADHD symptoms in regular cycling women [27]. Furthermore, one study indicated that estrogen levels in children with ADHD were similar to those in the control group [28].

No study has yet performed a comprehensive investigation of the associations between EDCs and gonadal hormones in children with ADHD. Prevalence and pathophysiology may be distinct between boys and girls with ADHD, so we hypothesized that EDCs and gonadal hormones influence susceptibility to ADHD. To clarify this, we conducted a cross-sectional study to examine whether the aforementioned EDCs and gonadal hormones demonstrated differential levels between ADHD patients and healthy controls in both boys and girls. We further examined hormones as intermediates in the analysis given the endocrine disruption nature of these chemicals.

## 2. Materials and Methods

### 2.1. Study Participants

For this study, we recruited children with ADHD who were treated in the outpatient Department of Child Psychiatry at Kaohsiung Chang Gung Children’s Hospital in Taiwan, whose parents and/or guardians rendered written consent forms, and who met the following criteria: (a) kids with clinical diagnosis of ADHD by a senior child psychiatrist based on the criterions of Diagnostic and Statistical Manual of Mental Disorders (DSM–5) [3,29]; (b) kids aged between 6 and 12 years old; and (c) kids who were medication-free or who have an existing diagnosis but were drug-free for at least 6 months. Some studies had revealed that ADHD medication may influence the levels of gonadal hormones [30,31]. To avoid the confounding effect of ADHD medication, we restricted ADHD cases to those with clinical diagnoses of ADHD but without ADHD medication. However, we excluded those patients who had a comorbid of autism spectrum disorder, intellectual disability, psychotic disorders, major depressive disorder, bipolar disorder, or neurological disorders.

Control subjects were recruited from the same geographic area as the ADHD patients. They were children who did not have ADHD or any other psychiatric disorders (such as autism spectrum disorder, intellectual disability, psychotic disorders, major depressive disorder, bipolar disorder, or neurological disorders). In total, we recruited for this study 98 boys and 32 girls with ADHD in addition to 42 boys and 26 girls as controls.

### 2.2. Laboratory Testing of Endocrine-Disrupting Chemicals and Sex Hormones

About 1.0 mL of urine sample was taken and enzymatically hydrolyzed and purified through solid phase extraction. The phthalate metabolites in the urine were then divided by reverse-phase ultra-performance liquid chromatography and identified by the electrospray ionization tandem mass spectrometry and quantified using an isotope internal standard curve method [32].

We purchased HPLC-grade ethyl acetate from Duksan Pure Chemicals and ammonium acetate (97.0% powder) from Merck. β-Glucuronidase (≥85,000 units/mL) from Helix pomatia (Type H-2) was obtained from Sigma–Aldrich (Merck KGaA, Darmstadt, Germany). Urine samples were embattled with 50 µL of internal standard (six types mixer each phthalate metabolite-13C12, 200 ng/mL) spiking solution, 1 mL of 2 M ammonium acetate buffer solution (1.54 g of ammonium acetate/10 mL HPLC-grade water), and 20 µL of β-Glucuronidase. Samples were incubated for 1 h at 37 °C, extracted twice with 4 mL of ethyl acetate, gently shaken a few times, then the organic layer was separated from the non-polar fat layer with a centrifuge at 4000 rpm for 15 min. We performed the chromatographic separation on a Shim-pack GIST C18 column (2.1 mm × 100 mm, 2 µm) from Shimadzu Co. Ltd. Target compound was performed with a Shimadzu 8050 Triple Quad liquid chromatograph mass spectrometer equipped with a Shimadzu LC-30 series HPLC system. The following EDCs were identified in this study: mono-methyl phthalate (MMP), monoethyl phthalate (MEP), mono-n-butyl phthalate (MnBP), monobenzyl phthalate (MBzP), monoethylhexyl phthalate (MEHP), methylparaben (MP), ethylparaben (EP), propylparaben (PP), butylparaben (BP) and bisphenol A (BPA). Limits of quantitation (LOQ): 0.8 ng/mL for MMP, 1.0 ng/mL for MEP, 0.8 ng/mL MnBP, 0.8 ng/mL for MBzP, 0.8 ng/mL for MEHP, 1.0 ng/mL for MP, 1.0 ng/mL for EP, 1.0 ng/mL for PP, 1.0 ng/mL for BP, and 1.0 ng/mL for BPA. Limits of detection (LOD) were ≤0.5 ng/mL for all compounds. The between-day imprecision (relative standard deviation) ranged from 3.6 to 8.7%, and the mean relative recoveries ranged from 75.2 to 121.5%.

Blood samples were collected from the participants in the morning after an overnight fasting to detect the serum levels of follicle stimulating hormone (follitropin, FSH), luteinizing hormone (lutropin, LH), estradiol, progesterone, testosterone, free testosterone, SHBG, and prolactin using an electrochemiluminescent immunoassay on a DPC Immulite 2000 XPi device (Siemens Healthcare Diagnostics, Tarrytown, NY, USA) [33]. Analytical Sensitivity: 0.1 mIU/mL for FSH; 0.05 mIU/mL for LH; 15 pg/mL (55 pmol/L) for estradiol; 0.1 ng/mL (0.3 nmol/L) for progesterone; 15 ng/dL (0.5 nmol/L) for testosterone; 0.02 nmol/L for SHBG; 0.5 ng/mL (10.6 mIU/L) for prolactin.

### 2.3. Clinical Measurements

To confirm the ADHD diagnosis based on the DSM-5 criteria, a senior psychiatrist interviewed those ADHD patients and control subjects. Moreover, an experienced child psychologist conducted the Wechsler Intelligence Scale for Children—Fourth Edition (WISC-IV). Parents and teachers of each patient were asked to fill out the SNAP-IV (Swanson, Nolan and Pelham Version IV) scale parent form and the SNAP-IV teacher form, respectively.

The WISC-IV is an individually administered and standardized test tool designed to determine the intelligence of kids aged 6–16 years [34]. It consists of 10 core subtests and five supplementary quizzes. The core subtests form the following four factor indexes: Verbal Comprehension Index (VCI), Perceptual Reasoning Index (PRI), Working Memory Index (WMI), and Processing Speed Index (PSI). The Full-Scale Intelligence Quotient (FSIQ) consists of 10 core subtests. A population mean of 100 and a standard deviation of 15 was for the four factor indexes and the FSIQ [34].

The SNAP-IV, a 26-item questionnaire, is used to assess the symptoms and severity of ADHD, which needs to be completed by parents or teachers [35]. These 26 items include 18 for ADHD symptoms (nine for inattention and nine for overactive/impulsive symptoms) and eight for oppositional defiant disorder (ODD) symptoms as defined by the DSM-5. Each item is scored from 0 to 3 on the Likert scale.

### 2.4. Statistical Analysis

The statistical software package SPSS, version 21.0 (SPSS Inc., Chicago, IL, USA) was used to analyze the data and present variables as either statistical means (standard deviation) or frequency. Two-tailed *p*-values of less than 0.05 were considered statistically significant.

We adopted the independent *t*-test to compare continuous variables between ADHD patients and healthy controls in both boys and girls. Endocrine-disrupting chemicals and gonadal hormones all demonstrated significant levels of positive skewness. We adopted arithmetic log transformations to establish approximate normal distributions for these levels.

We used the general linear model to assess the differences of endocrine-disrupting chemicals and gonadal hormones between ADHD and controls, setting age, urinary creatinine levels, and specific gravity as covariates. Partial correlation (age as the covariate) was used to determine the associations of EDCs with gonadal hormones and clinical measurements among boys and girls with ADHD, respectively.

## 3. Results

Included in our study (Table 1) were 98 boys with ADHD (average age: 8.5 years), 32 girls with ADHD (average age: 8.1 years), 42 control boys (average age: 8.7 years), and 26 control girls (average age: 9.2 years). Among the boys, no significant difference in age, height, or weight was found between the ADHD group and controls. Among the girls, the ADHD group was younger and had less body height and weight than the control group. Compared with the control groups, the ADHD group had poorer performance in all indexes of the WISC-IV. In inattention scores, hyperactivity/impulsivity scores, and oppositional scores of the SNAP-IV assessed by parents and teachers, the ADHD group showed higher severity than the control groups.

**Figure 1:**
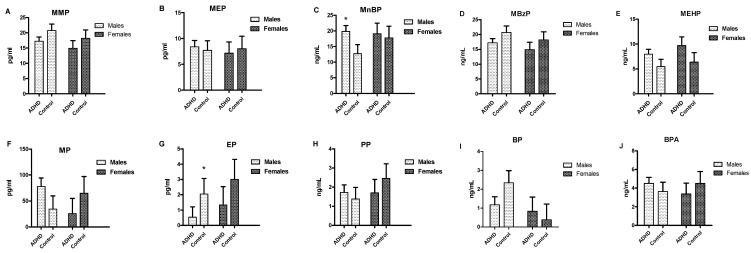
Serum levels of endocrine-disrupting chemicals (EDCs) in boys and girls among patients with ADHD and controls * *p* < 0.05.

**Figure 2:**
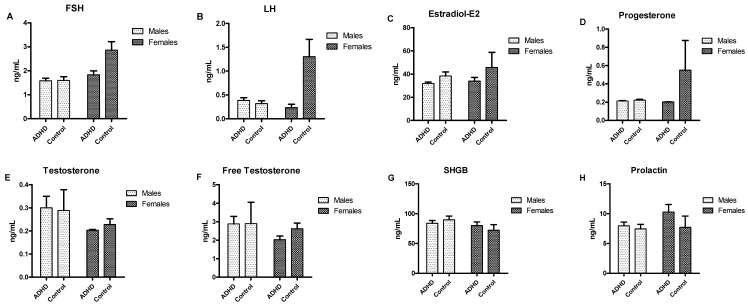
Serum levels of gonadal hormones in boys and girls among patients with ADHD and controls

Figure 1 shows the urine levels of the EDCs of the four participant groups. Compared to the boys in the control group, boys with ADHD demonstrated higher MnBP (*p* = 0.014) and PP levels (*p* = 0.032). However, we observed no significant differences regarding EDCs between the female with ADHD and control groups. Figure 2 displays the serum levels of the gonadal hormones of the four participant groups. No significant differences in testosterone, free testosterone, FSH, LH, estradiol, progesterone, or SHBG were found between the ADHD group and controls among either boys or girls.

The associations between EDCs and ADHD clinical measures and gonadal hormones were analyzed separately for ADHD boys (*n* = 98) and ADHD girls (*n* = 32). Among boys, we controlled for the confounding effect of age and found that urine MBzP levels (*r* = 0.572, *p* < 0.001) and MEHP levels (*r* = 0.406, *p* < 0.001) positively correlated with serum testosterone levels. And urine MEHP level was also positively correlated with serum prolactin level (*r* = 0.350, *p* = 0.001). Among girls, urine MEP levels were positively correlated with serum levels of LH (*r* = 0.646, *p* < 0.001), testosterone (*r* = 0.780, *p* < 0.001), and free testosterone (*r* = 0.407, *p* = 0.023). And urine BP level was also positively correlated with serum LH level (*r* = 0.490, *p* = 0.005).

Among ADHD boys, serum prolactin levels were negatively correlated with oppositional symptoms rated by parents (*r* = −0.266, *p* = 0.012), hyperactivity/impulsivity symptoms rated by teachers (*r* = −0.279, *p* = 0.008), and oppositional symptoms rated by teachers (*r* = −0.328, *p* = 0.002). Among girls with ADHD, serum LH levels were positively correlated with oppositional symptoms rated by teachers (*r* = 0.459, *p* = 0.014). Serum levels of FSH were positively correlated to hyperactivity/impulsivity symptoms rated by parents (*r* = 0.385, *p* = 0.043) and negatively correlated to inattention symptoms rated by teachers (*r* = −0.430, *p* = 0.022). The correlation matrix between EDCs, hormone levels, and ADHD characteristics are listed in Supplementary Tables S1–S6.

## 4. Discussion

The main findings in this study are that boys with ADHD had higher levels of MnBP and EP than control boys. Furthermore, several EDC chemicals were significantly associated with endocrine substrates. It is noteworthy that potential misclassification bias may exist when the hyperactivity/impulsivity symptoms rated by parents and teachers were used as the assessment tools. Since ADHD measures are not based on biomarkers, thus measurement errors concerning urinary chemical/hormone levels could potentially be non-differential with the health outcome.

Our results showed that MnBP and PP levels may play a role in boys’ susceptibility to ADHD. The results in this study were partially comparable to a previous review study [36]. The previous review study explored the relationship between various persistent organic pollutants, including exposure to PCBs, hydroxylated PCBs, polybrominated diphenyl ethers, dichlorodiphenyldichloroethylene, phthalates, BPA, and perfluorinated compounds and childhood neurodevelopmental outcome and demonstrated that exposure to environmental chemicals would affect neurodevelopmental outcome in children and had a gender-related vulnerability [36].

MnBP, a major metabolite of di-n-butyl phthalate (DBP), belongs to the group of phthalates. Phthalates serve as plasticizers to make plastics more flexible and are widely used in the production of polyvinyl chloride (PVC) products, food packaging, medical devices, cosmetics, and personal care products [11]. The finding of higher MnBP in boys with ADHD in this study was in line with that of previous studies [37,38,39]. EDCs manipulate sexual differentiation and may influence brain development during embryonic stage [40]. A study (76.7% male) showed that MnBP level was significantly higher in the ADHD group compared with the healthy control group [39]. A significant relationship between the urine MnBP levels and performance in continuous performance tests in school-age children was reported in another study [37]. In a Korean young population of 6–18-year-olds, MnBP level exhibited association with attention problem and the severity of hyperactivity [38]. Exposure to phthalates has three pathways: inhalation, ingestion, and dermal contact [41]. From this view, habitual behavior plays an important role in phthalate exposure, which may provide a possible explanation why not all ADHD participants in this study have higher MnBP levels.

EP (PP ?), a paraben, is a bacteriostatic and fungistatic agent used as a preservative in personal care products, pharmaceuticals, and foods [42]. Regarding exposure to parabens, developmental and reproductive toxicity related to parabens in vivo lacks physiological coherence and consistency [43]. Serum estradiol concentration significantly decreased in female Sprague-Dawley rat treated with EDCs chemicals during the juvenile-peripubertal period [44], while butyl-, methyl-, and propylparaben were associated with decreases in SHBG in pregnant women [45]. In an animal study, paraben inhibited antral follicle growth, altered the steroidogenic capacity of antral follicles, and increased the level of cell-cycle and apoptosis regulators of antral follicles [46]. Although we observed no differences in gonadal hormones between ADHD and control groups in this study, our findings suggest a link between EP and ADHD. Further research is warranted to determine the potential biological mechanisms underlying the observed associations of EP and ADHD.

BPA has been widely used in various consumer products, including plastics products, personal care products, and thermal receipt paper [47]. In our study, we could notice that BPA level was higher in ADHD boys than control, although it was not statistically significant. In an animal study, BPA exposure in postnatal stage could disrupt development of dendritic cells and neurotransmitter equilibrium in the rat hippocampus, which resulted in impaired spatial learning and memory [48]. Meanwhile, human studies have reported altered neurobehavior after BPA exposure during prenatal or childhood period, including aggressive behavior, ADHD, depression, and anxiety [49]. Despite the reduced number and heterogeneity of previous studies, the results have indicated that the effects may be sex-dependent [49]. More research is needed to confirm which gender is more vulnerable to BPA.

The results of this study showed no significant differences in testosterone, free testosterone, FSH, LH, estradiol, progesterone, or SHBG between the ADHD group and the control group, which is compatible with previous findings [26,28,50]. In a previous study [26], no significant differences in free testosterone or SHBG levels between ADHD patients and controls were observed. In addition, a previous study that evaluated serum estrogen and G protein-coupled estrogen receptor 1 (GPER) levels did not find an association between estrogen levels and ADHD [28]. Likewise, no associations were found between ADHD scores and free testosterone, estradiol, FSH, LH, or progesterone in women with polycystic ovary syndrome [50].

It is noteworthy that several EDCs chemicals were significantly associated with endocrine substrates. Among boys with ADHD, urine MBzP and MEHP levels were positively correlated with serum testosterone levels, and urine MEHP level was also positively correlated with serum prolactin level. Among girls, urine MEP levels were positively correlated with serum LH, testosterone, and free testosterone levels, and urine BP level was also positively correlated with serum LH level. In addition, levels of endocrine substrate were significantly correlated to ADHD characteristics. We suppose that EDCs had an adverse impact on the endocrine system, and further link to ADHD characteristics.

This study has several limitations. First, this study was a cross-sectional study, and the time effect of exposure and amount of EDC exposure on brain development were undetermined. EDC exposure at different life stages and differences in the amount may have led to different results. Moreover, because children affected by ADHD have a shorter life expectancy [51], ADHD children may expose themselves to EDCs due to their behavior, diets, and lifestyles. The exposure level will fluctuate depending on short-term exposure prior to sample collection e.g., diets, personal care product uses, etc. Similarly, gonadal hormones levels may be affected by child age, pubertal development, or the timing of collection. Whether the ADHD status or symptoms affected EDC exposure levels and hormonal functions thus leading to health risks should be explored in the future. Second, the sample size in the study was relatively small, particularly for the girl groups. Therefore, the statistical power in this study was limited in its ability to detect the impact of EDCs on different sexes within the ADHD and control groups. In addition, ADHD girls and controls had significantly different age and height. Thus, the selection of participants not only depends on ADHD status but also on factors related to exposure. In this case, the results could be subject to potential selection bias. Third, the statistical values were not corrected for multiple comparisons. All findings would become non-significant if correction for multiple comparisons is applied. Therefore, the robustness of the finding in this study should be verified in a larger sample. Despite these limitations, this is the first study to discuss the associations between MEP, MBzP, MP, EP, PP, BP, and ADHD and comprehensively investigate EDC levels and gonadal hormones with regard to the susceptibility of ADHD. Another strength is that all patients were drug-naïve and free from the confounding effect of medication.

## 5. Conclusion

The results suggest that MnBP and EP levels were higher in boys with ADHD than in their counterparts. Furthermore, several EDCs chemicals were significantly associated with endocrine substrates. EDCs may be involved in susceptibility to ADHD, particularly among boys. According to the results in this study, the possibility of an adverse impact of EDCs on neurodevelopment may exist. However, the results could be subject to potential selection bias, and the findings in this study should be interpreted with caution.

## Figures and Tables

**Table 1:** Comparisons of demographic data and psychopathology evaluations in boys and girls among patients with attention-deficit/hyperactivity disorder (ADHD) and healthy controls.

	Boys		Girls	
	ADHD (*n* = 98)	Control (*n* = 42)	ADHD (*n* = 32)	Control (*n* = 26)
Demographic data, mean ± SD				
Age, years	8.5 ± 1.7	8.7 ± 1.7	8.1 ± 1.3	9.2 ± 1.9
Height, cm	132.3 ± 12.4	133.3 ± 11.3	128.0 ± 11.6	136.9 ± 13.2
Body weight, kg	33.5 ± 13.9	31.0 ± 10.3	30.7 ± 13.8	34.2 ± 10.9
Comorbidities, *n* (%)				
ODD or conduct disorder	27 (27.8)	-	4 (12.5)	-
Tic disorders	15 (15.5)	-	1 (3.1)	-
WISC-IV, mean ± SD				
Full Scale Intelligence Quotient	98.6 ± 10.5	109.4 ± 15.1	96.9 ± 9.7	107.9 ± 11.9
Verbal Comprehension Index	102.4 ± 11.0	108.8 ± 12.2	101.8 ± 9.2	104.2 ± 10.5
Perceptual Reasoning Index	99.5 ± 12.7	110.4 ± 18.0	94.7 ± 10.6	109.9 ± 16.2
Working Memory Index	99.6 ± 11.5	108.5 ± 12.8	97.7 ± 10.9	107.7 ± 11.5
Processing Speed Index	94.0 ± 9.7	100.6 ± 12.1	94.9 ± 8.1	103.0 ± 11.9
SNAP-IV, mean ± SD				
SNAP-IV parent form (I)	16.5 ± 5.6	5.7 ± 6.3	17.1 ± 4.8	5.4 ± 5.5
SNAP-IV parent form (H)	15.6 ± 6.2	5.0 ± 5.6	12.7 ± 5.4	3.8 ± 5.6
SNAP-IV parent form (O)	12.7 ± 6.1	5.2 ± 5.1	10.8 ± 5.9	4.6 ± 5.1
SNAP-IV teacher form (I)	15.3 ± 5.3	4.9 ± 5.5	13.5 ± 7.0	4.2 ± 3.6
SNAP-IV teacher form (H)	13.7 ± 6.3	3.6 ± 4.0	7.7 ± 5.9	2.1 ± 2.5
SNAP-IV teacher form (O)	10.0 ± 6.2	2.0 ± 2.8	5.2 ± 5.0	1.3 ± 1.6
Urinary creatinine (mg/dL)	108.3 ± 39.0	104.1 ± 41.7	92.6 ± 29.1	107.7 ± 37.0
Urine specific gravity	1.01 ± 0.0	1.00 ± 0.0	1.00 ± 0.0	1.01 ± 0.0

Notes: data are expressed as mean ± SD or *n* (%); ODD, oppositional defiant disorder; SNAP-IV, the Swanson, Nolan, and Pelham—Version IV Scale for ADHD; WISC-IV, the Wechsler Intelligence Scale for Children—Fourth Edition; I, inattention scores; H, hyperactivity/impulsivity scores; O, oppositional scores. Z, Standardized Test Statistic using Mann–Whitney U Test.

## Supplementary Materials

The following are available online at https://www.mdpi.com/2305-6304/8/3/57/s1, Table S1: Correlations of EDCs in boys and in girls, Table S2: Correlations of gonadal hormone in boys and in girls, Table S3: Correlations of EDCs and levels of gonadal hormone for boys, Table S4: Correlations of EDCs and levels of gonadal hormone for girls, Table S5: Correlations of levels of gonadal hormone and symptoms of ADHD for boys. Table S6: Correlations of levels of gonadal hormone and symptoms of ADHD for girls.
