# Supplementary material for: Phthalates, Para-Hydroxybenzoic Acids, Bisphenol-A, and Gonadal Hormones’ Effects on Susceptibility to Attention-Deficit/Hyperactivity Disorder
Source: Toxics. 2020 Aug 13;8(3):57. doi: 10.3390/toxics8030057 (PMC7560246; doi:10.3390/toxics8030057)
Supplement: Supplementary file 1 [file toxics-08-00057-s001.pdf]

Supplementary Materials: Phthalates, Para–Hydroxybenzoic Acids, Bisphenol–A, and Gonadal Hormones' Effects on Susceptibility to Attention–Deficit/Hyperactivity Disorder

Ching-Shu Tsai, Wen-Jiun Chou, Sheng-Yu Lee, Min-Jing Lee, Miao-Chun Chou and Liang-Jen Wang

Table S1. Correlations of EDCs in boys and in girls.

	MMP	MEP	MnBP	MBzP	MEHP	MP	EP	PP	BP	BPA
**MMP**	–	0.146	0.025	0.020	0.053	0.008	−0.066	0.017	0.056	−0.018
**MEP**	0.332*	–	0.198*	0.113	0.157	0.128	−0.016	0.237**	−0.061	0.123
**MnBP**	0.332*	0.209	–	0.211*	0.278**	0.008	−0.006	0.087	−0.076	0.060
**MBzP**	0.222	0.305*	0.133	–	0.563**	−0.042	−0.068	−0.082	−0.079	−0.003
**MEHP**	0.448**	0.152	0.373**	0.473**	–	−0.054	−0.045	0.070	−0.072	−0.022
**MP**	0.341**	0.016	−0.034	−0.023	0.233	–	0.027	0.207*	−0.026	−0.024
**EP**	0.094	−0.018	0.164	−0.048	−0.013	0.023	–	0.199*	0.235**	0.036
**PP**	−0.036	0.121	0.186	0.004	0.033	0.021	0.325*	–	−0.005	0.054
**BP**	0.136	0.050	0.376**	0.010	0.281*	0.037	−0.057	0.074	–	−0.034
**BPA**	0.018	−0.015	−0.102	0.157	0.124	−0.038	−0.036	−0.159	0.190	–

Data presented in the Table are correlation coefficient evaluated using partial correlation (age as the covariate). The upper part of the correlation matrix are data in boys (shown in blue); the lower part of the correlation matrix are data in girls (shown in red). *$p < 0.05$; **$p < 0.01$ for significance level.

Table S2. Correlations of gonadal hormone in boys and in girls.

	LH	FSH	Testosterone	Free testosterone	SHBG	Estradiol–E2	Progesterone	Prolactin
**LH**	–	0.455**	0.241**	0.482**	0.011	−0.037	0.225**	−0.015
**FSH**	0.373**	–	0.052	0.086	0.021	−0.141	0.115	0.053
**Testosterone**	0.600**	−0.116	–	0.599**	−0.110	0.075	−0.039	0.089
**Free testosterone**	0.318	0.010	0.439**	–	−0.192*	0.050	−0.062	−0.007
**SHBG**	0.011	−0.091	0.174	−0.672**	–	−0.043	−0.045	0.007
**Estradiol–E2**	0.611**	−0.106	0.925**	0.359**	0.175	–	0.342**	0.020
**Progesterone**	0.573**	−0.136	0.979**	0.380**	0.194	0.934†	–	0.033
**Prolactin**	0.092	0.124	−0.014	0.136	–.179	−0.055	−0.031	–

Data presented in the Table are correlation coefficient evaluated using partial correlation (age as the covariate). The upper part of the correlation matrix are data in boys (shown in blue); the lower part of the correlation matrix are data in girls (shown in red). *$p < 0.05$; **$p < 0.01$ for significance level.

Table S3. Correlations of EDCs and levels of gonadal hormone for boys.

	MMP	MEP	MnBP	MBzP	MEHP	MP	EP	PP	BP	BPA
**LH**	−0.098	−0.004	0.055	−0.128	−0.056	0.082	−0.022	−0.022	−0.085	−0.001
**FSH**	−0.178	0.099	0.155	−0.019	−0.013	0.179	−0.021	−0.043	0.092	−0.022
**Testosterone**	0.106	0.030	0.065	0.572**	0.406**	−0.032	−0.037	−0.070	−0.072	0.018
**Free testosterone**	0.004	−0.020	0.060	−0.057	−0.008	0.028	−0.040	−0.057	−0.130	0.034
**SHBG**	−0.068	−0.065	−0.064	0.020	0.060	−0.083	0.044	0.042	0.047	−0.031
**Estradiol−E2**	0.043	−0.048	0.039	0.151	−0.123	−0.186	−0.064	−0.152	−0.004	0.005
**Progesterone**	−0.074	−0.093	−0.061	−0.185	−0.286**	0.148	−0.033	0.195	−0.007	−0.041
**Prolactin**	0.137	−0.060	−0.026	0.190	0.350**	−0.026	−0.132	−0.071	−0.039	−0.157

Data presented in the Table are correlation coefficient evaluated using partial correlation (age as the covariate). ***$p < 0.01$ for significance level.

Table S4. Correlations of EDCs and levels of gonadal hormone for girls.

	MMP	MEP	MnBP	MBzP	MEHP	MP	EP	PP	BP	BPA
**LH**	0.032	0.646**	0.283	−0.192	0.151	0.108	0.083	0.089	0.490**	0.159
**FSH**	−0.174	0.176	0.221	−0.284	−0.041	−0.007	−0.137	−0.035	0.149	0.138
**Testosterone**	−0.149	0.780**	−0.128	−0.131	−0.132	0.037	0.092	0.131	−0.082	−0.085
**Free testosterone**	−0.219	0.407*	0.038	−0.274	−0.203	0.226	0.152	0.164	0.176	0.262
**SHBG**	0.140	−0.114	−0.166	0.184	0.129	−0.265	−0.181	−0.135	−0.214	−0.223
**Estradiol−E2**	0.257	−0.063	−0.115	0.245	0.270	0.345	−0.060	−0.119	0.007	0.370*
**Progesterone**	0.234	−0.135	−0.034	−0.073	−0.203	−0.136	−0.052	−0.122	−0.094	−0.198
**Prolactin**	−0.004	0.083	−0.064	−0.084	−0.191	−0.162	−0.139	−0.152	0.023	−0.114

Data presented in the Table are correlation coefficient evaluated using partial correlation (age as the covariate). *$p < 0.05$; **$p < 0.01$ for significance level.

Table S5. Correlations of levels of gonadal hormone and symptoms of ADHD for boys.

	LH	FSH	Testosterone	Free testosterone	SHBG	Estradiol−E2	Progesterone	Prolactin
**Inattention (parent)**	0.035	0.111	0.029	−0.031	−0.039	0.062	0.082	−0.064
**Hyperactivity/Impulsivity (parent)**	0.025	−0.015	0.036	−0.046	0.028	0.033	0.117	−0.140
**Opposition (parent)**	−0.170	−0.117	−0.048	−0.072	−0.048	0.029	−0.065	−0.266*
**Inattention (teacher)**	−0.004	0.079	−0.057	0.054	−0.091	0.231*	−0.020	−0.189
**Hyperactivity/Impulsivity (teacher)**	−0.052	0.057	−0.042	−0.040	0.075	0.162	−0.079	−0.279**
**Opposition (teacher)**	0.005	−0.001	−0.058	0.137	−0.154	0.137	−0.117	−0.328**

Data presented in the Table are correlation coefficient evaluated using partial correlation (age as the covariate). *$p < 0.05$; **$p < 0.01$ for significance level.

Table S6. Correlations of levels of gonadal hormone and symptoms of ADHD for girls.

	LH	FSH	Testosterone	Free testosterone	SHBG	Estradiol−E2	Progesterone	Prolactin
**Inattention (parent)**	0.010	0.296	−0.088	−0.116	0.185	−0.277	0.321	0.172
**Hyperactivity/Impulsivity (parent)**	0.083	0.385*	0.053	0.098	0.029	−0.283	0.135	0.086
**Opposition (parent)**	0.198	0.295	0.128	0.020	0.130	−0.219	0.049	0.000
**Inattention (teacher)**	−0.209	−0.430*	−0.097	−0.158	0.259	0.164	0.157	−0.305
**Hyperactivity/Impulsivity (teacher)**	0.129	0.222	0.304	0.042	0.146	0.215	−0.119	−0.192
**Opposition (teacher)**	0.459*	0.143	0.267	0.261	−0.082	0.208	−0.245	−0.220

Data presented in the Table are correlation coefficient evaluated using partial correlation (age as the covariate). *$p < 0.05$; ** $p < 0.01$ for significance level
